# An Active Inference Account of Skilled Anticipation in Sport: Using Computational Models to Formalise Theory and Generate New Hypotheses

**DOI:** 10.1007/s40279-022-01689-w

**Published:** 2022-05-03

**Authors:** David J. Harris, Tom Arthur, David P. Broadbent, Mark R. Wilson, Samuel J. Vine, Oliver R. Runswick

**Affiliations:** 1 School of Sport and Health Sciences, College of Life and Environmental Sciences, University of Exeter, St Luke’s Campus, Exeter, EX1 2LU UK; 2 Division of Sport, Health and Exercise Sciences, Department of Life Sciences, Brunel University London, London, UK; 3 Department of Psychology, Institute of Psychiatry, Psychology, and Neuroscience, King’s College London, London, UK

## Abstract

Optimal performance in time-constrained and dynamically changing environments depends on making reliable predictions about future outcomes. In sporting tasks, performers have been found to employ multiple information sources to maximise the accuracy of their predictions, but questions remain about how different information sources are weighted and integrated to guide anticipation. In this paper, we outline how predictive processing approaches, and active inference in particular, provide a unifying account of perception and action that explains many of the prominent findings in the sports anticipation literature. Active inference proposes that perception and action are underpinned by the organism’s need to remain within certain stable states. To this end, decision making approximates Bayesian inference and actions are used to minimise future prediction errors during brain–body–environment interactions. Using a series of Bayesian neurocomputational models based on a partially observable Markov process, we demonstrate that key findings from the literature can be recreated from the first principles of active inference. In doing so, we formulate a number of novel and empirically falsifiable hypotheses about human anticipation capabilities that could guide future investigations in the field.

## Key Points

**Table:** 

Predictive processing approaches to the brain are becoming increasingly influential neuroscientific theories for understanding perception and action.
We outline how active inference, a predictive processing approach, may be a fruitful theory for understanding expert anticipation in time-constrained tasks.
A series of computational models were used to illustrate how active inference parsimoniously accounts for previous empirical findings of sport anticipation.

## Introduction

During tasks such as ball-striking sports, aviation or car driving, rapidly changing visual scenes may preclude the use of a purely reactive response strategy [1–3]. Consequently, effective performance depends on anticipating future outcomes [4]. Research into anticipatory behaviour has identified that during temporally constrained tasks, skilled individuals make better use of multiple sources of probabilistic information to anticipate future outcomes [5–9]. However, a number of fundamental questions remain about when different information sources become available, the relative weighting afforded to each source, and how they are integrated in an efficient and effective manner [6, 8, 10]. Answers to these questions may require looking beyond the traditional anticipation literature to more fundamental research into how the brain uses internal models to make predictions [11–14]. In the present work, we outline how active inference, a unifying account of how predictive models guide perception and action [15–17], can account for key findings in the sports anticipation literature and may provide a framework for future work in this area [14].

### Anticipation in Sport

Rather than waiting passively for stimulation, human brains are thought to continuously generate expectations about sensory input, future states of the body and the surrounding environment [11, 18]. Making predictions facilitates better information pick-up [19], helps to disambiguate the noise present in the environment and our neural system [20], and assists perception and cognition by pre-sensitising representations, leading to faster recognition and interpretation of stimuli [21]. The need for prediction is no more starkly illustrated than by the perceptual challenge of attempting to intercept a moving object, such as a tennis serve or baseball pitch [22]. Inherent visual processing delays mean that moving objects often cannot just be monitored. Instead, their future states must, in part, be predicted based on current states and internal models of likely trajectories [22–24]. This is the predictive challenge that faces elite athletes and has driven research interest into superior anticipation in time-constrained tasks.

Previous work on sporting anticipation has illustrated that effective predictions can be made by attending to sources of current sensory information that provide early cues to likely outcomes. These include postural information such as the non-kicking leg of a penalty taker in soccer [25], relative motion between players in team sports [26], ball flight motion [27] and even auditory cues from racquet-ball contact [28]. Predictions can be further improved by combining current sensory information with relevant probabilistic knowledge generated from contextual information, such as game score [29], or the playing tendencies [30] and location [31] of an opponent. Researchers have, however, called for a more detailed understanding of how various information sources are integrated, weighted and utilised over time [2, 6, 9, 10].

Initial research has supported the role of Bayesian probability computations in performing these information integration functions. Bayesian brain approaches contend that human perceptual computations are based on representing information sources as conditional probability density functions (i.e. a distribution of possible values with a mean and variance), which are then combined according to their certainty or reliability [32]. For example, in a handball penalty kick task, Helm et al. [33] found that trained novice participants integrated information sources (opponents’ kinematic information and action tendencies) according to their relative reliability, such that more informative action cues dominated over other ambiguous information. While this recent work provides compelling evidence that performers may employ Bayesian reliability-based strategies (for review, see Gredin et al. [8]), it has largely neglected to account for the dynamic interaction between the anticipator and the opponent [6]. This is partly owing to the frequent use of video stimuli in experiments, which have provided an important foundation for anticipation research, but allow very little interaction between the anticipator and the environment. Therefore, to begin to understand how more dynamic interactions between the anticipator and their surrounding environment shape anticipation, we require an alternative theoretical approach that is built on Bayesian inference principles but also explains more active and interactive anticipation-enhancing behaviours.

### Active Inference

Bayesian predictive processing models of the brain [15, 34] portray perception as a process of continually making and revising hypotheses about the most likely causes of any incoming sensory stimulation (i.e. the hidden states of the world that are not sensed directly). Within a predictive processing approach, hypotheses or beliefs about the likely causes of sensations (e.g. the object from which a certain light pattern originates) are constantly revised by the sensory prediction errors that occur when predictions do not fully account for sensations [35]. Predictions are based on an internal or ‘generative’ model of the world that encodes beliefs about uncertain environmental states and the hidden causal relationships that underpin dynamic sensory interactions [20, 34]. Crucially, internal models can be used to predict new sensory data (e.g. the feedback expected when executing an action [36]), or can work backwards to infer the hidden states that likely caused some observation [37] (known as model inversion).

Active inference is an extension of this predictive processing approach, applied to the use of action to minimise prediction errors. Active inference is rooted in the idea that the brain is a self-organising dynamical system (e.g. see Friston [20]) and must be understood as a product of its interactions with its surrounding environment. As a self-organising system, the brain interacts with the environment in such a way as to remain within certain preferred stable states (e.g. homeostasis). This is achieved by seeking to minimise prediction errors from the environment [20], with computationally surprising observations indicating the system has deviated away from a preferred stable state. Crucially, active inference proposes that surprisal can be minimised by either revising the generative model following a prediction error (i.e. to improve future predictions) [35] or by actively changing the world into the expected state—the exact element that has been missing from much of the sport anticipation literature (Table 1). Active inference, therefore, is the extension of predictive processing to the use of actions to minimise future surprisal [14].

**Table 1:** Definitions for active inference-related terminology

Dynamical system	A system whose state evolves over time according to a fixed rule, i.e. the values of key variables in a dynamical system can be modelled as functions of those same variables at earlier timepoints
Free energy	A measure derived from information theory that provides an upper bound on the surprisal of some data. Under simplifying assumptions, free energy is equivalent to the sum of prediction errors
Posterior	The updated belief after combining a prior belief and some newly observed information
Precision	The expected confidence or reliability of a piece of information, formally, the reciprocal of the variance of its probability distribution
Prior	A probability distribution that would express one’s beliefs about a hidden state or parameter
Self-organisation	A process whereby overall order arises from the local interactions between parts of a system
Sensory prediction errors	The computed difference between predicted and actual sensory input
Surprisal	A measure of the unexpectedness of a stimulus. Mathematically, the negative log-probability of the event outcome

Optimising predictions about the world (to minimise prediction error) requires integrating observed information with prior beliefs, according to their reliability or precision, in a continuous and dynamic manner. The implication for sport anticipation is that prior probabilistic knowledge (i.e. contextual information) and current sensory information will both be used wherever they are informative but weighted according to the expected precision of each. For instance, Gray and Cañal-Bruland [38] demonstrated that when baseball batters were provided with prior probabilistic information about the likelihood of different pitch types, they altered swing kinematics in response to cue reliability. Crucially, information about the prior probability of the pitch types was relied upon more heavily when online visual information was occluded to a greater degree (i.e. was known to be less precise). However, further empirical scrutiny is required to examine whether these context-sensitive actions represent Bayes-optimal processing in the human sensorimotor system.

### Present Work

**Fig. 1:**
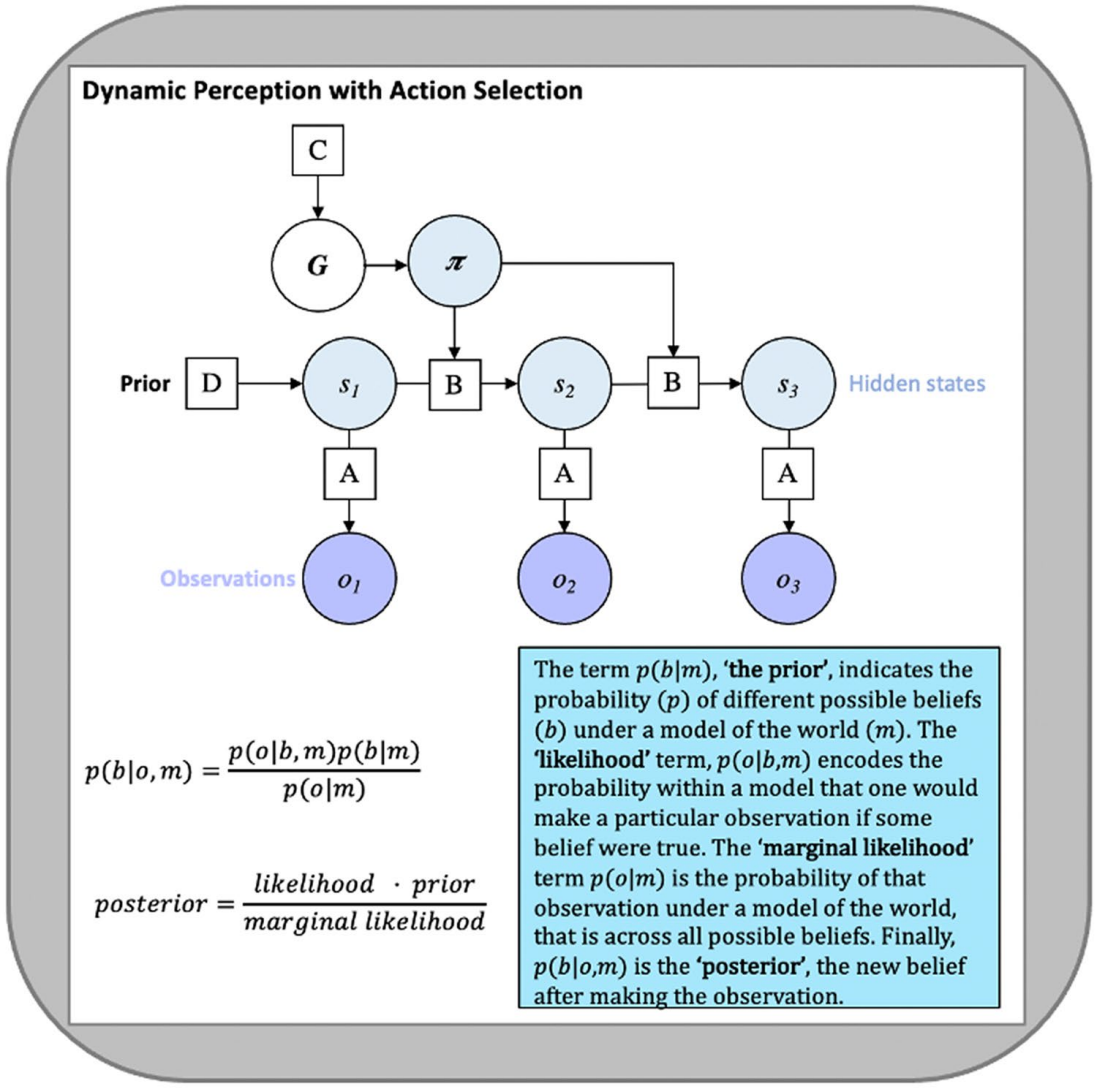
Bayesian network representation of the partially observable Markov decision process model. Note. The network (top) depicts the vectors and matrices that are used to model anticipatory behaviour. Circles (‘nodes’) within the network correspond to the following variables: s = hidden state to be inferred, o = observations, π = possible action choices, G = expected free energy. Squares indicate factors mediating conditional relationships, which take the form of matrices in the model: A = likelihood mapping between hidden states and observations (i.e. (*o*_τ_|*s*_τ_)), D = priors over hidden states (i.e. (*s*_1_)), B = state transition matrices encoding beliefs about how hidden states evolve over time (i.e. (*s*_τ+1_|*s*_τ,_)). Observation and state subscripts indicate timepoints within a trial (*τ*). When *τ* > 1, the B matrix from τ − 1 acts as an empirical prior (as D vector at *τ* = 1). C = matrices encoding prior preferences over particular observations and behavioural states. Arrows connecting nodes indicate dependencies between variables. The lower elements of the figure depict Bayes theorem, which specifies how a prior belief is updated via observed instances to form the posterior belief

The extensive sport anticipation literature has recently been reviewed and summarised within the Model of Information Use During Anticipation in Striking Sports (MIDASS) by Runswick and colleagues [6]. Runswick et al. synthesise a range of empirical anticipation literature to outline how various information sources are integrated during skilled anticipation in striking sports, such as tennis or baseball. In doing so, they identify seven key principles for how prior probabilistic knowledge and current sensory cues are used; some have extensive empirical support, while others require future testing. Here, we used these seven principles as a guide to the main empirical results that a plausible theory of anticipation would need to account for. Therefore, we outline each of the principles identified in the Model of Information Use During Anticipation in Striking Sports framework and simulate the corresponding Bayes-optimal anticipatory behaviour within a computational model (see Fig. 1). We used this as a way of discussing the core principles of active inference and how they can be applied to sport anticipation. While there has been much empirical research in this area, it perhaps lacks an underpinning neuroscientific theory. Our aim is not to provide detailed models of specific tasks or to prove these seven principles, indeed, they are already supported by extensive empirical work, instead, we aim to demonstrate the utility of active inference for understanding why and how these patterns of anticipatory behaviours emerge. As a development of existing theory, we also describe how an active inference scheme leads to additional predictions about anticipatory behaviour. These new predictions, generated from synthesising frameworks from sport science and neurocomputational psychology, present new hypotheses that can be examined in future empirical work.

## Methods

Computational models that simulate the Bayes-optimal behaviour of hypothetical observers have been used widely in the active inference literature and applied to areas such as language, interoception and visual illusions [39–41]. The aim of this simulation modelling approach is to demonstrate what Bayes-optimal behaviour is like (under plausible constraints), how it relates to observed human behaviour and how active inference can account for empirical findings. Crucially, this is not a mechanistic claim that humans employ exact Bayesian inference [42, 43]. Rather, it is an illustration of how human behaviour may approximate Bayesian inference under different environmental constraints. Bayesian modelling approaches have previously been criticised as unfalsifiable on the basis that the free parameters of such models enable them to fit almost any behaviour. However, this is not a uniquely Bayesian issue, as computational models of all varieties are similarly flexible [42]. More importantly, the point of this approach is to better understand the perceptual problem and generate testable hypotheses about how human observers solve that problem, rather than for the models themselves to provide empirical evidence.

Here, we used this simulation approach as a method to demonstrate the basic principles of active inference and how they may play out during a sport anticipation task. We present a simple scenario, commonly used in previous empirical research, in which a performer must intercept a moving ball that has a number of different possible speed and flight profiles [27, 38]. This could apply to the delivery of a fastball or curveball in baseball, or a normal speed vs a slower ball in cricket (where unexpectedly slower deliveries are often missed or shots are mistimed). We model the situation where an observer is required to anticipate and intercept a ball that has one of two possible trajectories, referred to as ‘normal’ and ‘change-up’ hereafter. Accordingly, we also draw on examples from cricket and baseball to illustrate many of the effects.

### Model Simulations

The partially observable Markov decision process (POMDP) modelling approach used here is based on a Bayesian generative model of perception derived from the Markov decision process formulation of active inference [17]. The POMDP models assume the Markov property in the sense that the generative model operates entirely on local information, as all previous states are encoded within current beliefs. Within the POMDP model, prior beliefs about hidden states such as contextual information (*s*_*τ*_: which ball is more likely?) take the form of categorical probability distributions that are updated through integrating beliefs about past states (*s*_*τ*−1_: which ball have I just faced?), current sensory information (*o*_*τ*_: what ball do kinematic cues indicate?) and beliefs about future states (*s*_*τ*_ _+1_ : which ball is likely to come next?). When the observer influences outcomes with their actions (*π*), transitions between states (*B*_*τ*_) become conditional on actions, which means future prediction errors are being minimised through action (i.e. active inference). See Table 2 for a summary of the computational terms.

**Table 2:** Description of computational elements for generative model

Model variable	General definition	Model-specific definition
*τ*	Timepoint within a trial	In both scenarios (‘simple’ and ‘trade-off’), there are three timepoints: start state, seeing the cue (sensory information), and finally anticipating the ball and observing the outcome
*o*_*τ*_	Observable outcomes at time τ	1. Ball with normal trajectory 2. Ball with change-up trajectory
*s*_*τ*_	Hidden states at time τ	1. Normal ball 2. Change-up ball
A matrix (*p*(*o*_*τ*_|*s*_*τ*_))	Matrix encoding beliefs about the relationship between hidden states and observable outcomes (i.e. the likelihood)	Beliefs about the relationship between observed sensory information and hidden state of ball trajectory. Additionally, beliefs about the likelihood of being correct
B matrix (*p*(*s*_*τ*+1_|s_τ_))	Matrix encoding how beliefs about states will evolve over time	Encodes the prior belief that either a normal or change-up ball would occur on each trial (i.e. contextual information)
D vector (*p*(*s*_τ = 1_))	Matrix encoding beliefs about initial hidden states	Starting prior belief about the prevalence of normal and change-up balls (i.e. contextual information)
*π*	Action policy probabilities	Prior distribution over policies (anticipate normal or change-up), made up of preferences for particular policies (not used here) and the expected free energy (*G*) of each policy
*G*	Expected free energy	Evaluates the value of anticipating normal or change-up based on the preferred and expected observations for each policy
*C*	Preferred outcomes	A matrix encoding the ‘reward’ or ‘loss’ value (i.e. prior preference) for each possible outcome

An observer’s beliefs about the utility of action policies (i.e. its expected value) depends on the negative log evidence it expects to obtain for each policy, that is, the expected free energy (denoted G). G is calculated from expected outcomes (and their precision) but also from preferred outcomes (or prior preferences, specified in the C matrix). The POMDP model proceeds to estimate the posterior over states via an optimisation routine (based on gradient descent) that aims to minimise free energy in the model. This routine determines what a Bayes-optimal integration of probabilities would be, given the starting conditions, and therefore the actions of the observer.

We present models of two general anticipation scenarios: a ‘simple scenario’ and a ‘trade-off scenario’. In both, the observer is required to anticipate and respond to the ‘normal’ or ‘change-up’ ball. In the simple scenario, the observer receives sensory information in the form of a cue early in each trial, which provides information about the most likely outcomes (akin to observing early kinematic cues from the opponent delivering the ball). However, the informational properties of the cue and the observer’s prior beliefs (i.e. contextual information related to whether ‘normal’ balls are more or less likely) are manipulated across the different examples to illustrate the effect this has on anticipation. In the trade-off scenario, the sensory information is only observable later, relative to action initiation, such that waiting for the new information incurs a cost to the value of the outcome. This is modelled by a lower expected reward value (i.e. a reduced preference for the outcome), to simulate the impairment that can occur in dynamic tasks if actions are initiated too late. Consequently, this scenario trades off the benefit of early predictions with greater certainty about outcomes. See Smith et al. [44] for further explanation of the mathematical formalism of these models, or the additional detail in the Electronic Supplementary Material [ESM] (https://osf.io/8bpkr/) or the MATLAB code available from the ESM (https://osf.io/vuy8e/).

## Results

In this section, we present the simulations of anticipatory behaviour when Bayes-optimal observers acted to intercept a series of ‘normal’ or ‘change-up’ balls with variation in the reliability of current sensory cues (e.g. ball flight, postural information) and prior probabilistic knowledge (e.g. previous encounters with the pitcher). In the models, we refer to ‘free energy’, an information theoretic concept that relates to the surprisal of the data (see Table 1 for definitions). For current purposes, free energy can be understood as a prediction error, and the selections of actions by the observers in the model are driven by the need to minimise future or expected free energy with their action choices. We modelled a simple scenario where the observer could make an anticipatory action (e.g. movement towards the ball) that would enhance their chances of success, provided they had anticipated correctly. Simple adaptations to the starting conditions of the models generated a range of anticipatory behaviours that corresponded closely with previous empirical findings and the main anticipation principles outlined by Runswick et al. [6].

### Principle 1

“Both contextual and current sensory information can influence anticipation performance directly, but this effect is neutral (chance level) until knowledge of the relationships between information sources and event outcomes is developed by a performer” (Runswick et al. [6], p. 204).

This principle is based on studies that have shown task experts, with their extensive domain knowledge, are better able to make use of both prior knowledge of context and current sensory information during anticipation [45, 46]. Active inference explains this effect through the more developed state of the expert performer’s generative model, which is better able to make predictions from the context and interpret sensory information [37]. Without existing knowledge, prior beliefs about the context or sensory information will equate to a flat probability distribution (e.g. Fig. 3A) and will have little influence on action selection [32].

**Fig. 2:**
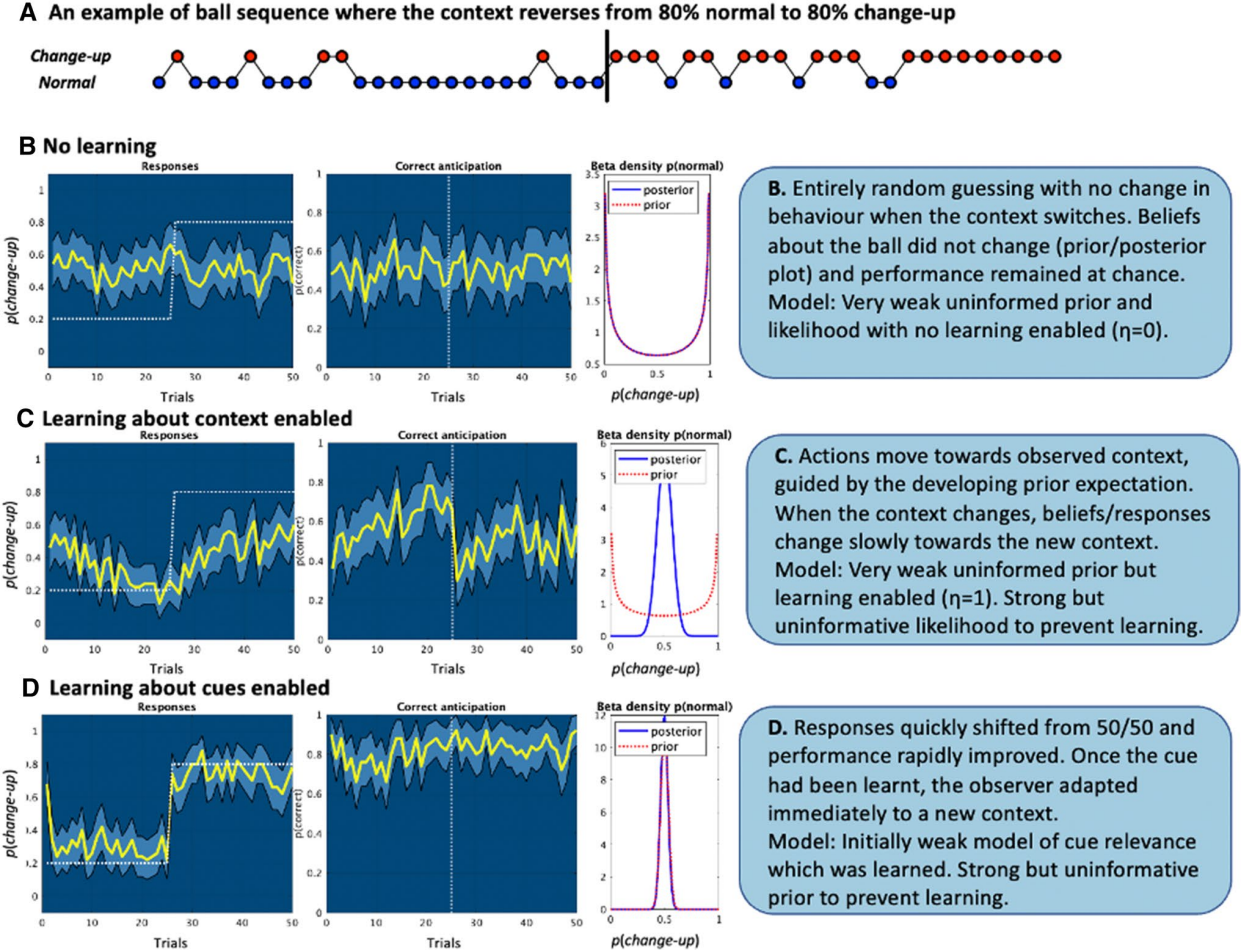
Partially observable Markov decision process simulations of action choices (left), anticipation success % (middle), and prior and posterior beliefs about *p*(change-up) [right] for 50 observers across 50 consecutive trials. Note: plots on the left represent the mean (yellow line) proportion of actions that were made to anticipate the normal ball (light blue 95% confidence intervals). The white dotted line represents the veridical probability in the left column, and the shift point in the middle column. Beta density plots in the right column, which describe the density (*y*-axis) of a continuous probability distribution defined over the interval [0, 1] (*x*-axis), indicate the mean prior and posterior beliefs over the 50 simulated observers

The POMDP model simulations (Fig. 2) illustrated that in our simple anticipation scenario, preventing learning about either prior context or the relevance of sensory information (any early cues) led to random guessing about the most appropriate actions by simulated observers. When prior context or sensory cue learning was enabled, performance improved over time as informative beliefs developed. Results (Fig. 2) show that using either the sensory information or the known context can aid performance, but only after the generative model has been developed, as has been observed from empirical work [45, 46]. The simulations also suggest that prior context and current sensory cues may differentially affect the speed with which observers are able to respond to environmental changes (e.g. the contrasting effect of reversing the frequency of normal and change-up balls [i.e. context reversal] in Fig. 2C, D).

### Principle 2

“Contextual information is available before current sensory information. Earlier judgements are therefore based predominantly on context. Information available later (e.g. postural cues or ball-flight) will be used to confirm, update, or override original judgements” (Runswick et al. [6], p. 204).

**Fig. 3:**
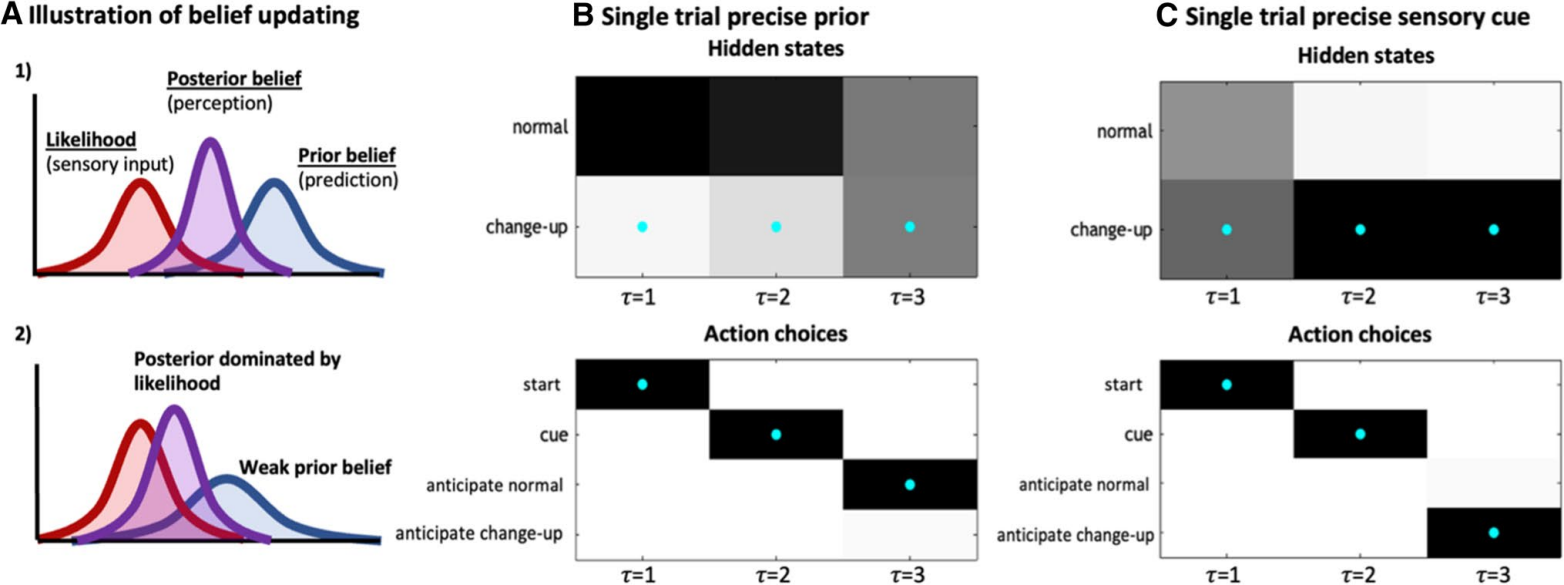
Illustrative probability distributions during belief updating (**A**), and plots of fluctuating beliefs within single trials from a partially observable Markov decision process model (**B**, **C**). Note: plots in **B** and **C** show the strength of posteriors over hidden states at the end of a single trial (i.e. what the observer believed at each timepoint *τ*, when at the last time point *t*): *τ* = 1 is the starting state, *τ* = 2 is when the sensory information is observed, and *τ* = 3 is after the observer has seen the true outcome. In the plots, darker squares indicate higher probability values for beliefs about states (top) or likelihood of selecting actions (bottom), and the cyan dots denote the true states. The ‘hidden states’ box displays the observer’s beliefs about the ball and the ‘action choice’ box shows the most likely (darker squares) and actual (i) anticipatory actions the observer selected. In B, the observer began with a strong prior belief about contextual information (that normal was more likely), but the subsequent sensory information was uncertain. Hence, observing sensory information that indicates the change-up was coming was insufficient to revise their belief, and they still acted to anticipate the normal ball. Even after clearly observing the change-up ball, their belief about the true contextual information remained uncertain (grey squares at *τ* = 3 in B). In **C**, the observer begins with a less precise prior belief that normal is the true context and receives more precise sensory information. Consequently, beliefs are revised at *τ* = 2 when the sensory information is observed and the observer acts to anticipate the change-up ball. In effect, under a strong prior belief about contextual information (**B**) the sensory information had little impact on beliefs and action choices, but when the current sensory information was more certain (**C**) beliefs were adjusted during the trial and a different action was selected

As active inference is based on Bayesian probabilistic integration, the relative influence of contextual information and current sensory information at different timepoints is a direct result of the expected precision of the respective information sources, i.e. how reliable it is believed to be [8, 15, 32]. Hence, sensory information that is evaluated as more reliable or precise will overwhelm an imprecise prior belief about contextual information to dictate the posterior, and subsequently action selection (see Fig. 3A).

Figure 3B, C illustrate this effect for two single-trial simulations where beliefs about the more likely hidden state (normal or change-up) are updated across timepoints within a single trial according to the precision of each informational source, resulting in different action selections. These simulations correspond closely with many empirical findings in the sport anticipation literature [33, 38, 47, 48]. For instance, Runswick et al. [46] found participants relied heavily on game context information when the delivery of a cricket ball was occluded at an early stage, but increasingly employed current sensory information for later occlusions, as the precision of the kinematic cues (e.g. bowler’s action) increased (see also [47]).

### Principle 3 [and 4]

“When information is congruent [incongruent] with the event outcome, this will enhance [impair] anticipation performance and the greatest positive [negative] impact on performance will occur when all sources of contextual and sensory information are congruent [incongruent] with the event outcome” (Runswick et al. [6], p. 205).

### Principle 6

“The opponent can deliberately manipulate information sources to his/her advantage to decrease anticipation accuracy. This effect occurs by deliberately developing incongruent relationships between contextual information, sensory information, and event outcome” (Runswick et al. [6], p. 206).

These three principles, which all relate to the congruency of available information with the true outcome, describe how knowledge of prior contextual information and current sensory information can influence performance either positively or negatively, depending on their truth [49]. The congruency of information sources can vary naturally (e.g. an attempted curveball of a baseball pitcher still goes straight) or can be deliberately manipulated by an opponent as an attempt to gain an advantage (e.g. when the pitcher disguises their curveball). For instance, following a training phase in which they learned the likely throwing direction of an opponent, handball goalkeepers were more accurate at anticipating throwing direction when it was congruent with their training experience, and vice-versa [30]. Similarly, Jackson et al. [50] demonstrated this congruency effect for deceptive ‘stepover’ actions in soccer, which were more likely to deceive the observer when congruent with known high-probability outcomes. Within active inference and predictive processing schemes, beliefs are the product of integrating information sources according to their precision [32]. Hence, if either is incongruent with the true outcome, but believed to be accurate, expectations about likely outcomes will initially be skewed by the inaccurate information source, until they are learnt to be imprecise.

**Fig. 4:**
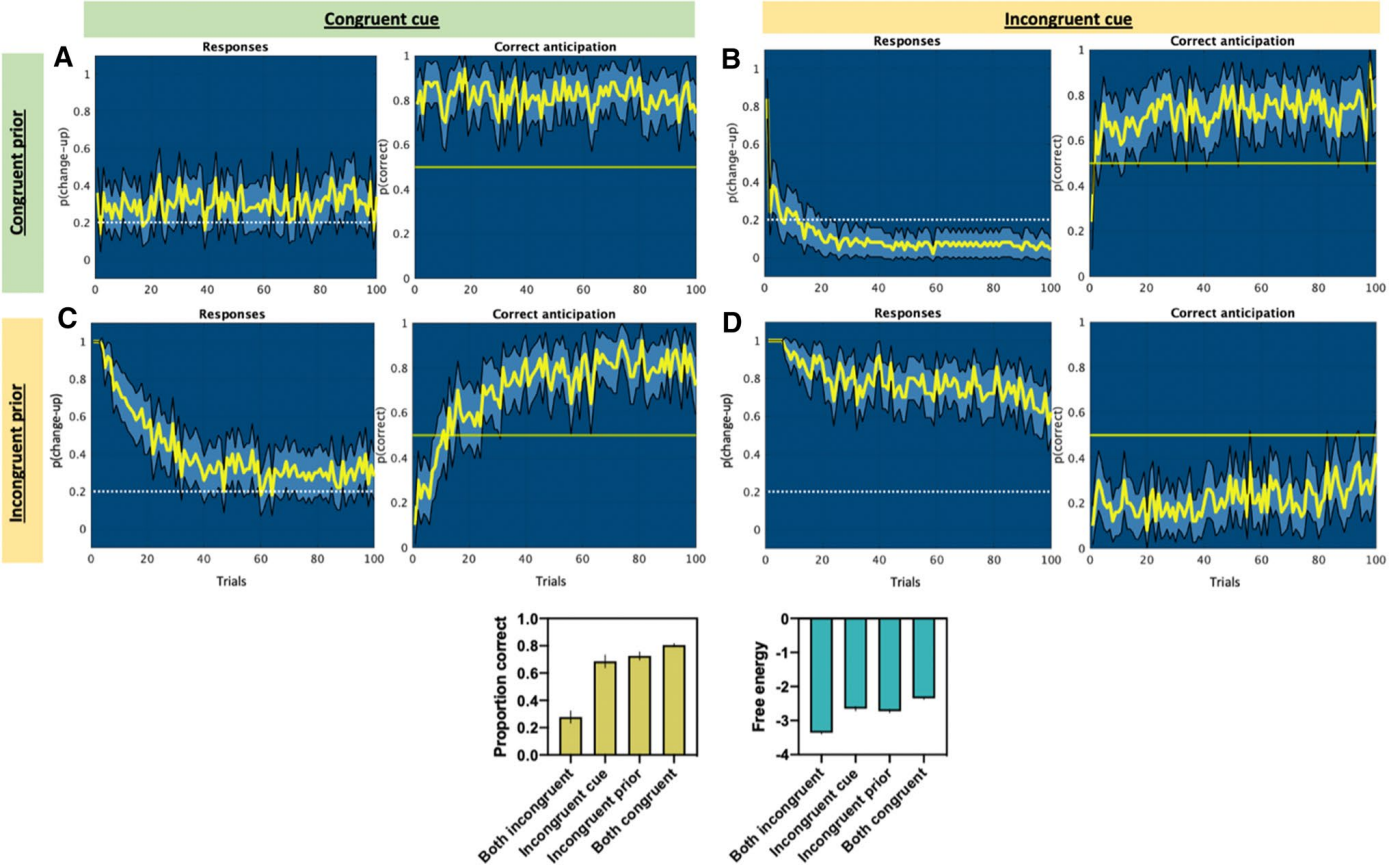
Partially observable Markov decision process model results showing anticipatory action choices (left in each panel) and performance (right in each panel) for congruent and incongruent information sources using 50 simulated observers over 100 consecutive trials in the simple scenario. Note: the left-hand plot of each panel **A–D** shows the proportion of actions that were made to anticipate the normal ball (white dotted line represents the veridical probability), and the right-hand plot of each panel shows the mean proportion of successes (yellow horizontal line is chance performance). The plotted yellow line indicates the mean (light blue 95% confidence intervals). When both information sources are congruent, performance is good and responses are close to the true outcome (**A**). When the sensory information source is incongruent but the contextual information is congruent (**B**), the observer is initially biased by the incorrect sensory information. However, as the observer learns that the sensory information is either unreliable or might even specify the opposite outcome, action selection comes to be dominated by the more reliable contextual information and observers rigidly stick to predicting the normal ball only. When the contextual information is incongruent (**C**), and more precise than the sensory information, responses are initially heavily biased towards the wrong outcome, which appears to be the optimal choice, but this is quickly unlearnt (although depends on the strength of the contextual information). Finally, when both information sources are incongruent with the true state (**D**), performance is worse than guessing but begins to improve as the observer unlearns their beliefs about the contextual and sensory information through observing the true outcomes. Indeed, their performance will continue to improve over further trials as they learn the true mappings of the information. Where there was at least one incongruent source, performance was initially very poor, but improved over trials as the incongruent information was learnt to be unreliable (i.e. low precision) and came to be down weighted in posterior beliefs. This learning was much slower when both information sources were incongruent (as in Jackson et al. [50]]). Incongruent sensory information appeared to cause a more permanent disruption, as beliefs about contextual information were quickly adjusted (**C**) but incongruent sensory information caused a lasting decrement (**B**). The lower plots summarise the overall performance and the total free energy in each condition (where more negative is greater)

The POMDP simulations (see Fig. 4) using the same scenario as for principle 1 illustrated that anticipation was indeed more accurate when observers were provided with a congruent source of information, and even better when both online sensory and prior contextual information were congruent, as observed in studies of anticipation [51, 52]. The results also suggest that incongruent information will negatively affect anticipation initially, but the effects should diminish over time as the generative model adapts, or as skill levels increase. From the perspective of the opponent, deceptive cues may, therefore, only result in a temporary advantage when other reliable information is also available.

### Principle 5

“Congruent and incongruent information can act simultaneously; the overall anticipation performance will depend on how the anticipator prioritises information and the reliability of information sources and the point of time in the anticipation process” (Runswick et al. [6], p. 206).

This principle relates closely to those above (3/4 and 6) but highlights the importance of how different informational sources are weighted during anticipation. Active inference and related Bayesian approaches identify expected precision as the key determinant of how informational sources will be weighted [15, 32, 34]. Either prior contextual information or current sensory information can be prioritised depending on their reliability, which is realised through modulating the strength of neuronal error signals [35]. Studies of anticipation have shown that performers do not always use all available information [53, 54]. For instance, in a simulated handball task, Helm et al. [33] found that observers relied more heavily on prior contextual information when current sensory information was more uncertain because of disguised actions.

**Fig. 5:**
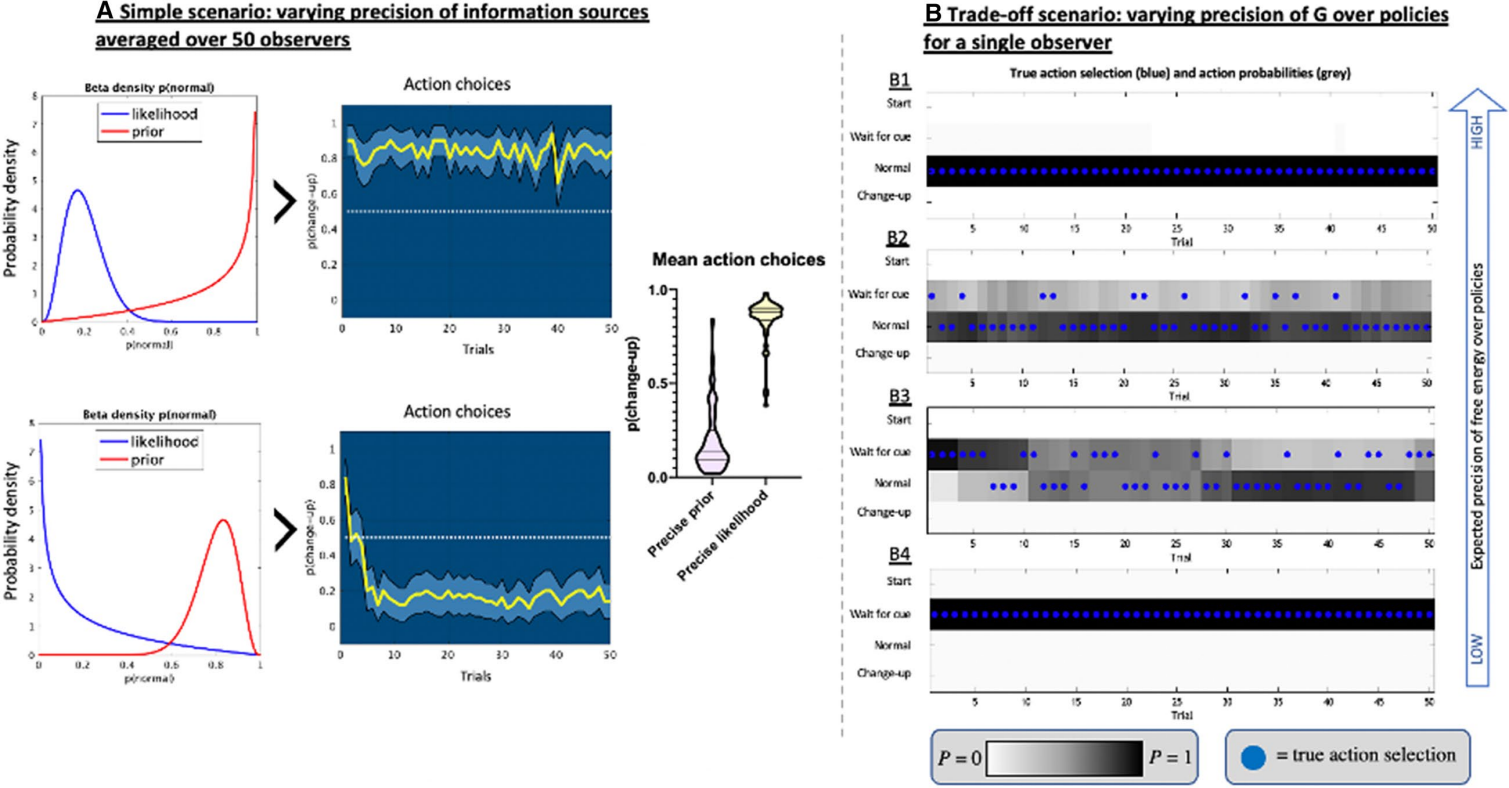
Partially observable Markov decision process simulations illustrating the effect of the precision of prior beliefs about contextual information and current sensory information (**A**) and the precision of beliefs about action outcomes (**B**). Note: in the simple scenario (**A**), the true occurrence of normal and change-up balls was set at 50/50. Fifty observers held a prior belief about the contextual information that ‘normal’ balls were more likely, but current sensory information always indicated that ‘change-up’ was more likely than ‘normal’. The ratio of beliefs about the conflicting sources remained the same across the two conditions (four to one), but the precision changed, such that in the top scenario the stronger beliefs about the informational value of the sensory information dominated action choices, and vice versa in the bottom left. Beta density plots (left) show the strength of the prior and posterior for the two simulations, and the action choices plots show the proportion of anticipatory actions favouring the normal vs the change-up ball. For the trade-off scenario in (**B**), the plots show the actions that were chosen by the observer (blue dots) along with the probability of those actions being selected (i.e. the action probability; darker squares indicate higher probability). Here, prior beliefs about contextual information or sensory information do not vary, but the precision of beliefs about expected free energy for different action policies (G in the network model; Fig. 1) decreases over the four conditions (B1–B4). When beliefs were more certain (B1), the observer chose to anticipate early to maximise the reward value of their action. However, when the precision of G was lower (B4), the observer was information seeking and chose to wait for later sensory information. In the middle panels (B2 and B3), the observer shows a mix of waiting and predicting early, as they learn the most effective strategy

The POMDP simulations in Fig. 5A illustrate this effect for the simple scenario when contextual information and sensory information were placed in conflict, i.e. prior beliefs about contextual information favoured one ball type and current sensory information indicated the converse. Here, observers aligned their anticipatory behaviour with whichever source was believed to be more reliable, much as in the single-trial simulations in Fig. 3. In addition to integrating information sources according to expected precision, active inference also predicts that action selection will be based on the expected precision of free energy over action policies. This means actions will be favoured when the observer is able to accurately predict their effects, but habitual responses (i.e. reliable actions that are low in uncertainty) will be favored when the precision of alternatives is low. As a result, the observer may not always act precisely in line with their beliefs about the most likely outcome, they will also act according to how well they can predict the effects of their action. Simulations in Fig. 5B illustrate this effect within a POMDP model of the trade-off scenario, where the observer opted to anticipate early when they were certain about the effects of their actions but waited for additional sensory cues when they were unsure.

### Principle 7

“The responder can deliberately manipulate situations to his/her advantage and increase anticipation accuracy” (Runswick et al. [6], p. 207).

An issue that has so far received limited attention in the sports anticipation literature is how performers can deliberately manipulate situations to enhance predictions by increasing the likelihood of certain outcomes. This lack of focus on the interaction between performer and environment may be due to the traditional experimental paradigm of responding to video clips, where action choices have no influence on outcomes. Indeed, to move research in this area forward, it would be useful to incorporate more continuous interactions between the performer and environment in experimental designs, whether that is through more interactive video-based paradigms, virtual reality or field work. From an active inference perspective, how actions interact with the environment is a crucial component of anticipation because action is one way to resolve uncertainty [16, 55]. In essence, uncertainty can be minimised by: (i) choosing to wait for additional information or (ii) acting in a way that biases the situation towards more predicted outcomes [56].

At one level, performers can manipulate the situation by adapting a motor action to avoid prediction errors. For instance, in a study of cricket batters, Sarpeshkar et al. [57] report that when balls with a straight trajectory were interleaved with those with a curvilinear (i.e. swinging) trajectory “the uncertainty created by ball-swing significantly altered movement behaviour …, with batters moving closer to the ball and hitting it earlier” (p. 98), thereby minimising prediction error. Alternatively, actions can be used to deliberately bias the behaviour of another performer, such as making space for an opponent to move into (to confirm the observer’s predictions) or closing down space to force them into a decision (disambiguating between uncertain hidden states). In summary, the interacting role of the performer and environment during skilled anticipation is not well understood, but may be one of the key methods that performers resolve uncertainty and maximise their predictive abilities. While the modelling of complex multi-person interactions is beyond the scope of the current paper, the principles of prediction error minimisation, maximisation of preferred outcomes and precision weighting of information that we have described will also guide action choices in these situations.

### Additional Hypotheses About Anticipatory Behaviour Derived from Active Inference Modelling

#### Reward Value and Loss Aversion

During active inference, the calculation of expected free energy is driven not only by uncertainty minimisation (i.e. epistemic value) but also maximising ‘reward value’ (i.e. pragmatic value) [55, 58]. As active inference is a belief-based system, reward refers to achieving preferred outcomes and observations, rather than some objective value function as conceptualised within reinforcement learning (see Sajid et al. [56]). This means actions are information seeking under uncertainty, but reward seeking when confidence in beliefs is already high (i.e. when there is no more uncertainty to resolve) [58]. Consequently, anticipatory behaviours will be influenced by preferred, as well as likely, outcomes. Supportive findings have been observed in motor control tasks [59], as well as sporting anticipation, where cricket batters’ estimates of ball direction were biased towards the stumps [52], which could reflect the weighting of predictions by the potential loss incurred if the ball hit the stumps (see also Gredin et al. [60] in soccer).

**Fig. 6:**
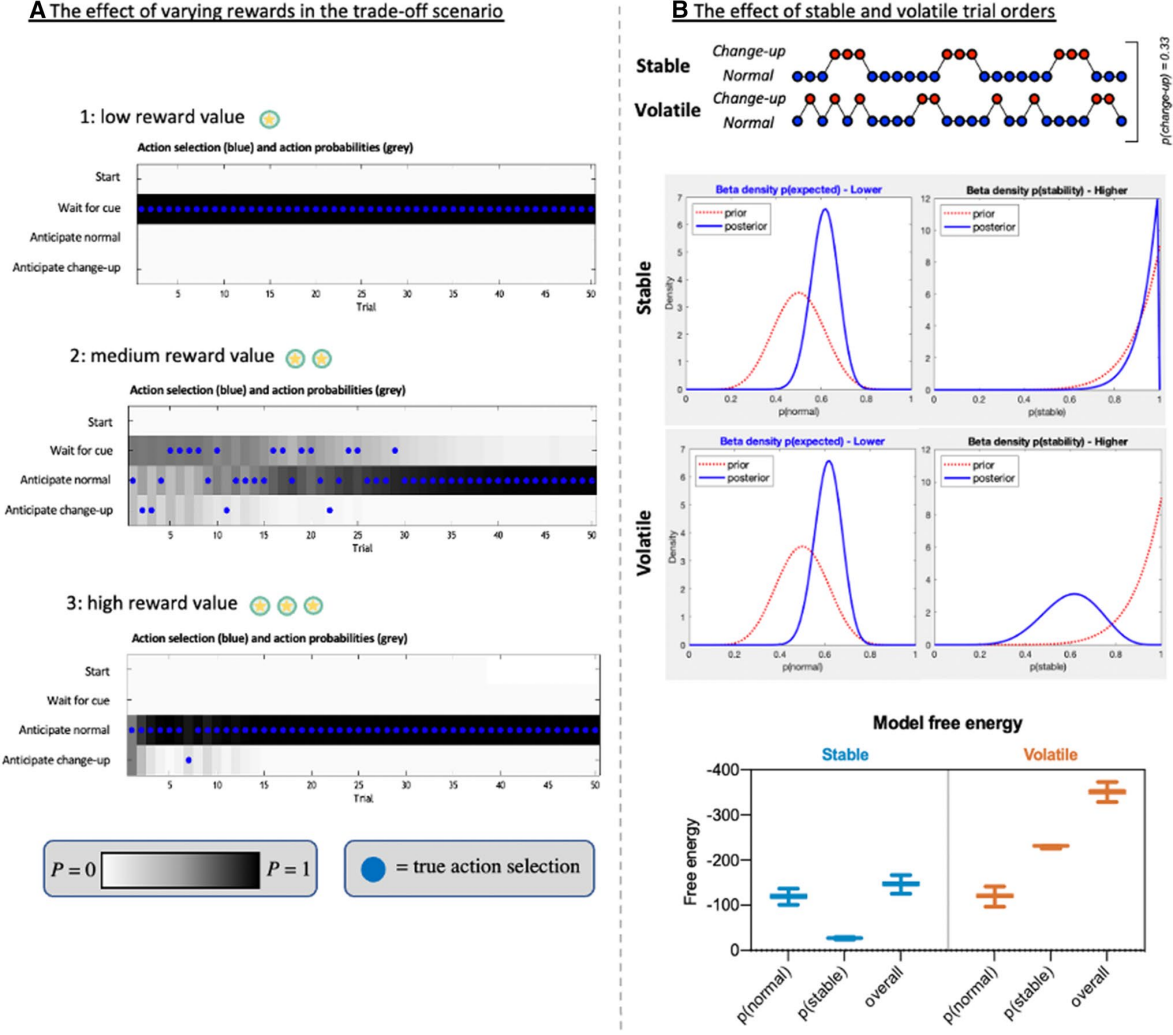
Single observer simulations across trials in the trade-off scenario (**A**) for low (A1), medium (A2), or high (A3) reward values, and the effect of a volatile environment on free energy (**B**). Note: in the trade-off scenario in (**A**), each plot shows the actions that were chosen by the observer (blue dots) along with the probability of those actions being selected (i.e. the action probability; darker squares indicate higher probability). The observer has the option to wait for the sensory information or execute an early anticipatory action. In all three scenarios, the observer starts with a weak prior belief that the contextual information is 50/50 (true context is 80/20) and veridical knowledge of the relationship between the sensory information and hidden states. The observer is clearly information seeking in A1, when rewards are low, and reward seeking in A3 when rewards are high. Crucially, in A2, the observer initially waits for the sensory information (and guesses a little), but switches to predicting the normal ball after they have learned about the 80/20 contextual information. **B** Illustrates the effect of a volatile trial order on free energy in the simple scenario. Here, a ‘hierarchical’ model was used to illustrate the effect of contextual volatility. In addition to perceiving normal and change-up balls as depicted in the model in Fig. 1, a higher level was added that recognised the stability of the wider context (i.e. how changeable the trial order was). Hence, the probability of ‘normal’ and ‘change-up’ was encoded at the lower level while the changeability of the environment was encoded at the higher level. When comparing the predictable (stable) with the unpredictably changeable (volatile) trial orders, observers (*n* = 50) had equivalent beliefs about the likelihood of change-up balls (note similar posteriors in the ‘lower’ plots) and similar levels of free energy, as the marginal likelihood of normal/change-up balls was matched. However, expectations about stable contextual information (i.e. sequences of normal/change-up balls) at the higher level produced high levels of free energy when trial order was unpredictable. Under active inference, these greater prediction errors should lead to a greater weighting of recent information when making decisions in a more uncertain environment (see Arthur and Harris [63] for an example)

Simulations of the trade-off scenario with varying reward values (see Fig. 6) illustrate this effect of preferred outcomes on action selection, where lower reward values led to waiting for the sensory information (resolving uncertainty; Panel A) but higher reward led to riskier early predictions (seeking reward; Panel C). Figure 6B illustrates how observers may be information seeking initially and wait for later onset sensory information but search out preferred outcomes once they are more certain in their ability to make predictions from contextual information alone. In sporting terms, this could equate to initially conservative swing selection by baseball batters to ensure a hit against an unpredictable pitcher, compared to seeking out higher rewards (but at greater risk) when they become more familiar with the pitching style (i.e. looking for home runs). The weighting of such rewards will therefore depend on individual preferences for particular outcomes and tendencies toward reward seeking or loss aversion.

#### Changing Probabilistic Contexts

Another factor that has received limited attention in the anticipation literature is the changeability of probabilistic contextual information. The simulations presented here, and in previous studies in this area [38], have mostly examined changes in the marginal probability of different outcomes (e.g. a more or less likely fast ball in baseball). This is known as expected uncertainty; a form of uncertainty that arises from probabilistic relationships that exist in the world (e.g. the outcome of a dice is more uncertain than a coin toss) [61]. However, probabilistic relationships between a stimulus and an outcome can also be more, or less, changeable. This is known as unexpected uncertainty or volatility [61, 62]. Active inference predicts that unexpected shifts in probabilistic relationships creates additional uncertainty, which leads to greater weight being placed on more recent information. Simulations in Fig. 6B illustrate results from a hierarchical POMDP model where beliefs about the stability/volatility of the environment (i.e. how changeable trial order sequences are) are encoded at a higher level, while beliefs about the marginal likelihood of normal/change-up balls (contextual information) are encoded at a lower level. Here, expectations of stable contextual information related to trial order produced high levels of prediction error (free energy) when that expectation was violated, which should induce additional uncertainty about predictions. This effect has recently been illustrated in an interceptive motor task [63] where anticipatory eye movement profiles were adjusted according to the volatility of ball elasticity changes over time. A functionally increased learning rate was also observed when recent contextual information was more changeable. While these effects are likely to be subtle in comparison to changes in marginal probabilities, the changeability of probabilistic contextual information may be an important route for future work in anticipation.

## Discussion

Two broad sources of information, prior contextual information and current sensory information, enable performers to predict future outcomes in dynamic tasks when processing latencies prevent a purely reactive response strategy [5, 6, 8, 9]. However, key questions remain about how these sources of information are integrated and when each is prioritised [10]. Here, we have presented a series of computational models that not only provide a framework for answering these questions, but can also be used to generate new, theoretically driven hypotheses for research in this area. This modelling approach demonstrates the effectiveness of active inference for explaining the neurocomputational mechanisms behind the anticipatory behaviours observed in previous empirical work. These statistically driven conceptions of human behavior could unify wide-ranging theories of sporting performance, in a manner that is both biologically plausible and potentially valuable for applied practitioners.

The simulations demonstrated that the key principles arising from the skilled anticipation literature, as recently described by Runswick et al. [6], were accounted for by simple active inference models. This was largely achieved by varying the precision of available information sources (e.g. Fig. 5). We were also able to demonstrate the negative effect that incongruent information, from prior contextual information or current sensory information, would have on anticipation performance and how the reliability of such information can be learned and un-learned (e.g. Figs. 3, 4). It is important to note that the models presented are generic models that could be applied to numerous anticipation (or other decision-making) scenarios, which illustrates the flexibility and applicability of this approach to many behaviours. Crucially, these results illustrate that the key empirical findings about skilled anticipation fit neatly within an active inference framework.

As discussed, active inference is based on the assumption that individuals seek to maximise the predictive power of their model of the world, via minimising prediction error (free energy) [20, 55]. One way to do so, is to act on the world so as to minimise the surprisal of observations, such as hitting a ball earlier to minimise the possibility of a deviation [57], or waiting to collect more information before acting (as in Fig. 6A). This idea can significantly expand research into skilled anticipation to investigate how actions are used to maximise predictions, an issue that has been largely overlooked to date [6]. Moreover, this principle may also help us to understand other aspects of expertise. For instance, predictive visual behaviours are also an ‘action’ that aims to minimise surprisal through information sampling that either confirms predictions or disambiguates between possible outcomes [64]. An additional benefit of active inference is that it flexibly accounts for individual differences in anticipation that have been observed [65] via different prior beliefs and subjective expectations of precision. Therefore, adopting an active inference approach in future work could make a considerable contribution to our understanding of anticipation and sporting expertise more generally. Specifically, there are several reasons to suggest active inference can be a useful framework for future work:

1. **Key Findings in the Skilled Anticipation Literature Are All Direct Predictions of Active Inference Accounts of Perception and Action** Not only is active inference able to retrospectively account for many of the key findings in this area (as we have shown), but someone with no knowledge of the anticipation literature could most likely predict these findings if equipped with only a knowledge of the first principles of active inference. The true test of this approach, however, will be whether subsequent predictions derived from active inference (e.g. as we outline above) continue to be supported.
2. **Active Inference is Biologically Plausible** Active inference is not just a higher level conceptual framework, it is also realised in neuronal encoding [15]. Predictive coding models that describe how the sensory cortex makes inferences about sensory inputs [15, 34] can be implemented using simple computational elements and therefore could plausibly be performed by biological networks of neurons [66]. Indeed, there is growing evidence that populations of neurons enact the type of belief updating described in active inference and predictive coding theories [35] as well as modulate neuronal signal strength (gain) in proportion to signal precision [15, 66]. Those adopting a strong active inference position have even suggested that the organisation of descending connections in the motor cortex indicates that movements are not the result of motor commands, but are the fulfillment of motor predictions [35, 67].
3. **People Behave in a Qualitatively Bayes-Optimal Way in Many Tasks** A primary source of support for Bayesian brain approaches comes from empirical findings that illustrate human observers act in a manner consistent with Bayes-optimal models of behaviour [32]. For instance, during sensorimotor control [36], visual illusions [68] and associative learning [69], humans are near optimal in weighting the uncertainties that characterise prior beliefs and sensory inputs.
4. **Active Inference Provides a Unifying Framework and Generates Testable Predictions** Work by Friston and colleagues [15, 16, 58] extending predictive coding into the domain of action has created a deeply unified account of perception, cognition and action. An account that is rooted in the idea of the brain as a self-organising dynamical system and that highlights the importance of person-environment interactions [11]. However, perhaps the most compelling argument for adopting an active inference approach is that it provides clear testable predictions (especially when based on computational models), which is an ideal starting point for hypothesis testing. Outlining testable predictions was the primary aim of the Model of Information Use During Anticipation in Striking Sports [6], but the extension to active inference places these predictions on a more formal footing. As Griffiths et al. [42] note, the power of Bayesian frameworks may not even lie in their ‘truth’ but their potential for generating new predictions.
  An important caveat for the present approach is that the correspondence of computational models with observed data does not necessitate that observed behaviours were generated from the same computations. Indeed, Bayesian models of the brain do not necessarily claim that the human perceptual system is ‘optimal’, nor that it calculates probabilities in the same way as a computational model [42]. Rather, human behaviour may be Bayes-optimal with respect to a number of assumptions or constraints [11].

## Conclusions

In summary, we have demonstrated that active inference can account for various well-established findings within the sport anticipation literature via a few simple principles (e.g. prediction error minimisation and precision-weighted inference). While previous work has suggested anticipatory information may be integrated in a Bayesian manner [8], active inference extends this idea to the use of actions that minimise uncertainty and fulfil expected and/or preferred outcomes. This generates supplementary predictions: that anticipatory behaviour will be influenced by outcome preferences and a changing probabilistic context. Indeed, studies have begun to show how very specific predictions derived from active inference readily emerge during dynamic anticipation tasks [63]. Crucially, researchers do not need to adopt modelling approaches to work within this framework; a successful application of active inference would be to generate and test new hypotheses about skilled anticipation that are driven by fundamental theories about the predictive brain.

## Declarations

### Availability of data and material

All relevant data are available online from: https://osf.io/vuy8e/.

### Code availability

The code is available online from: https://osf.io/vuy8e/.
